# RNA-seq analyses of multiple meristems of soybean: novel and alternative transcripts, evolutionary and functional implications

**DOI:** 10.1186/1471-2229-14-169

**Published:** 2014-06-17

**Authors:** Lei Wang, Chenlong Cao, Qibin Ma, Qiaoying Zeng, Haifeng Wang, Zhihao Cheng, Genfeng Zhu, Ji Qi, Hong Ma, Hai Nian, Yingxiang Wang

**Affiliations:** 1 State Key Laboratory of Genetic Engineering and Collaborative Innovation Center for Genetics and Development, Institute of Plant Biology, Center of Evolutionary Biology, School of Life Sciences, Fudan University, 200433 Shanghai, China; 2 Institute of Biomedical Sciences, Fudan University, 200032 Shanghai, China; 3 State Key Laboratory for Conservation and Utilization of Subtropical Agro-bioresources, College of Agriculture, South China Agricultural University, 510642 Guangzhou, China; 4 Guangdong Sub-Center of National Soybean Improvement Center, South China Agricultural University, 510642 Guangzhou, China; 5 Institute of Biodiversity Sciences, Fudan University, 200433 Shanghai, China

**Keywords:** Soybean, RNA-seq, Transcriptome, Novel transcriptional regions, Alternative splicing, Meristem, Transcription factors

## Abstract

**Background:**

Soybean is one of the most important crops, providing large amounts of dietary proteins and edible oil, and is also an excellent model for studying evolution of duplicated genes. However, relative to the model plants *Arabidopsis* and rice, the present knowledge about soybean transcriptome is quite limited.

**Results:**

In this study, we employed RNA-seq to investigate transcriptomes of 11 soybean tissues, for genome-wide discovery of truly expressed genes, and novel and alternative transcripts, as well as analyses of conservation and divergence of duplicated genes and their functional implications. We detected a total of 54,132 high-confidence expressed genes, and identified 6,718 novel transcriptional regions with a mean length of 372 bp. We also provided strong evidence for alternative splicing (AS) events for ~15.9% of the genes with two or more exons. Among them, 1,834 genes exhibited stage-dependent AS, and 202 genes had tissue-biased exon-skipping events. We further defined the conservation and divergence in expression patterns between duplicated gene pairs from recent whole genome duplications (WGDs); differentially expressed genes, tissue preferentially expressed genes, transcription factors and specific gene family members were identified for shoot apical meristem and flower development.

**Conclusions:**

Our results significantly improved soybean gene annotation, and also provide valuable resources for functional genomics and studies of the evolution of duplicated genes from WGDs in soybean.

## Background

Legumes are one of the three largest families of flowering plants, have diverged from a common ancestor around 50 million years ago (mya), and are major players for biological nitrogen fixation with important contributions to agricultural systems [1]. Soybean [*Glycine max* (L.) Merr.] is the most important crop among legumes, providing ~70% dietary proteins and ~30% edible oil [2]. Soybean has 20 pairs of chromosomes with a predicted genome size of 1,115-Mb [3] and is a paleopolyploid with two lineage-specific whole genomic duplications (WGD). The most recent WGD in soybean history occurred at about 13 million years ago (mya) [4], more recent than those in the history of the model plants *Arabidopsis* and rice. The recently sequenced soybean genome with 950 megabase (Mb) (~85% of the estimated total) of assembled sequences has revealed the presence of many thousands of recent paralogs due to WGD [4], making it an excellent model for study the evolution of duplicate genes.

The genome sequences allowed the annotation of over 66,000 genes, including 46,430 that were designated as high-confidence genes, and ~20,000 that were predicted bioinformatically with lower confidence [4]. Recent transcriptome data provided evidence that soybean has a total of 55,616 transcripts [5]. The relatively recent WGD and tandem duplications (TD) have resulted in a genome with ~75% of the genes being members of multi-gene families [4,6,7]. In particular, among the 46,430 high-confidence genes, there are 15,632 groups of 2–6 close paralogs, including tandemly repetitive genes, while 15,166 other genes are single copy [4]. A recent study updated the duplicated genes to 17,547 pairs/groups, 8910 of them are pairs driven from the latest WGD [8]. Furthermore, soybean genome has 38,581 repetitive elements occupying 59% of the genome, which covers most types of the plant’s transposable elements [9]. However, the gene annotation in the soybean genome is still incomplete, and can be further improved by using information from genome-wide information of gene expression, including detection of novel transcribed regions and alternative splicing events.

The recent development of high-throughput RNA sequencing (RNA-seq) technologies has greatly improved sensitivity of transcriptomics and allowed detection of transcripts without a priori gene models [10-12]. RNA-seq has been applied extensively and successfully to explore genome-wide expression patterns, to identify novel transcripts, to detect alternative splicing events and trans-splicing RNA, in organisms from yeast to human [13-16]. Transcriptomics have also been performed extensively in the model plants *Arabidopsis* and rice, at the level of specific tissues and even single cell types, and for identification of novel transcribed regions and splicing patterns [17-22]. It has also been applied increasingly in other plant species, such as *Zea m*ays [23], wheat [24], *Fragaria vesca*[25], as well as soybean [5,8,26,27]. However, the current knowledge about soybean transcriptome is still incomplete. For example, many predicted genes in the soybean genome are not yet supported by expression information; also, relatively little is known about the patterns of alternative splicing events in soybean. In this study, we conducted RNA-seq for 11 soybean tissues and obtained large datasets for discovery of novel transcriptional regions and splicing transcripts, tissue preferentially or differentially expressed genes, transcription factors, conservation and divergence in expression patterns between duplicated gene pairs from recent whole genome duplications, as well as for functional implications by comparative transcriptome analyses.

## Results and discussion

### RNA-seq reveals ~ 54,000 transcriptionally active genes in soybean

To analyze the soybean (*G. max*) transcriptome as we had previously done for *Arabidopsis* and *zebrafish*[21,28,29], we collected 11 tissues from soybean, including root tip, hypocotyl, cotyledon, callus, shoot apical meristem at 6, 17 and 38 day stage (referred to as SAM6D, SAM17D and SAM38D for convenience), as well as the axillary meristem (referred to as AM), inflorescences before and after the meiotic stage (referred to as IBM and IAM, similar to the *Arabidopsis* inflorescences at stages 1–9 and 9–12, respectively), and open flower (referred to as OF), and obtained from 111 to 326 million reads of ~50 bp for each sample, with ~30-50 times more data than previous RNA-seq studies in soybean [5,30]. Among them, 52.3%-71.6% of the reads were mapped to the *G. max* reference genome [4], ~90% of the mapped reads matched annotated soybean genes (in Additional file 1: Figure S1a and in Additional file 2: Table S1). Furthermore, the genic distribution of reads showed that 75% of mapped reads corresponded to exons, while the remaining reads were distributed among introns (10%), intergenic regions (7%) and the splice junctions (8%) (in Additional file 1: Figure S1b and in Additional file 2: Table S2). Therefore, our RNA-seq provides high-quality data for further exploration of the soybean transcriptome.

**Figure 1:**
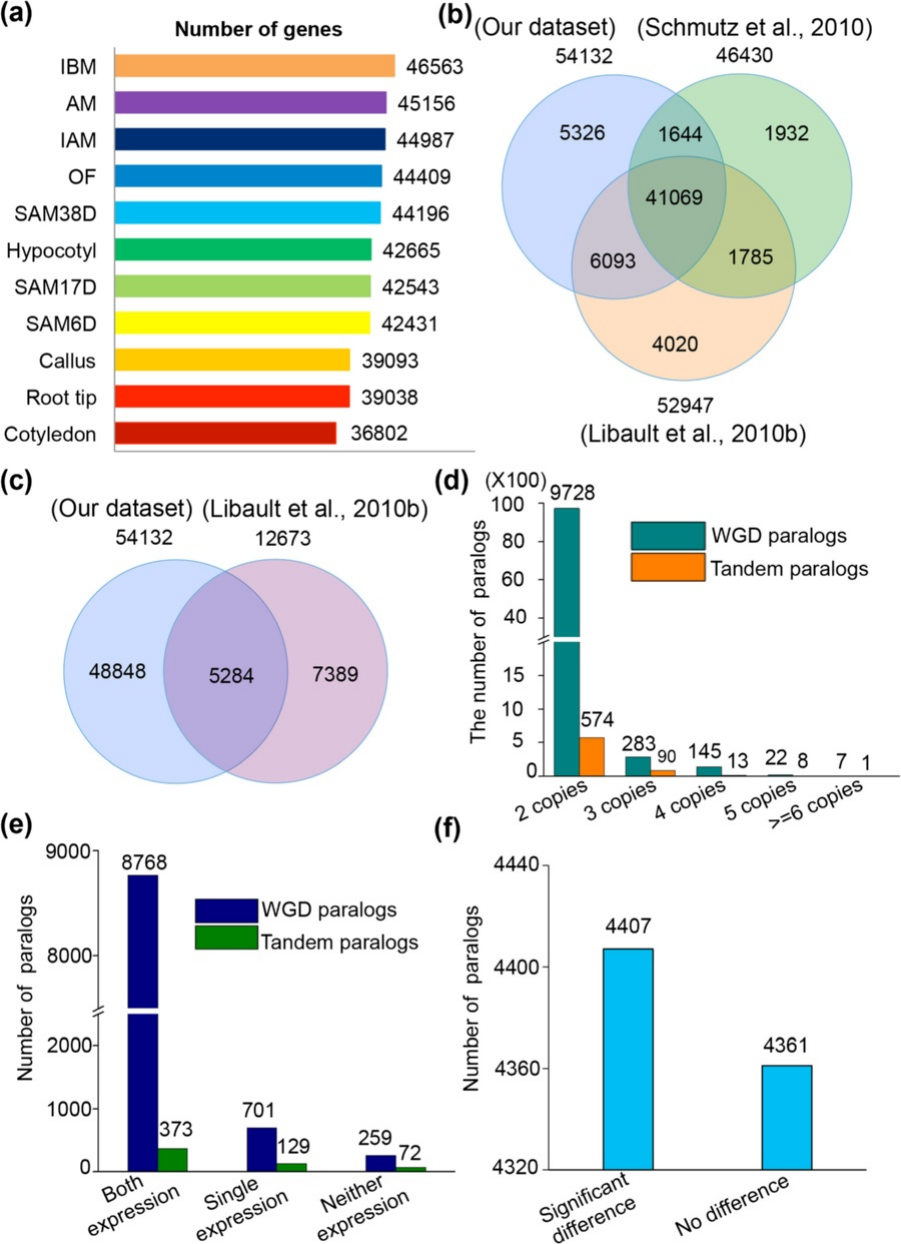
**Analyses of the detected genes and duplicated genes in 11 tissues. (a)** Number of genes detected in each of the 11 tissues. SAM6D, SAM17D and SAM38D refer to the shoot apical meristem at 6, 17 and 38 day after germination, respectively; IBM and IAM refer to inflorescence before and after meiotic stage; OF: open flower; AM: axillary meristem. **(b)** Comparison of the 54,132 detected genes with the 46,430 high-confidence genes (Schmutz et al. [4]) and the previously reported 52,947 transcripts in soybean (Libault et al. [30]). **(c)** Comparison of the 54,132 detected genes with the previously designated 12,673 pseudogenes in soybean (Libault et al. [30]). **(d)** Distribution of the WGD and TD paralogs in 2–6 copies based on the predicted 66,210 genes in soybean genome. **(e)** Distribution of 9,728 WGD paralogs and 574 TD paralogs in pairs from the 54,132 detected genes. **(f)***T*-test analysis of the significant difference of expression levels between two paralogs in the 8,768 detected paralogs.

To estimate the number of genes that are expressed in the examined soybean tissues, we first normalized the gene expression value using a variation of the RPKM method (*R*eads *P*er *K*ilo-base of mRNA length per *M*illion mapped reads) [13,31,32], and distinguished reliable signals of gene expression from the background noise of experiments by comparison between expression level of genes and intergenic regions (in Additional file 1: Figure S2, see Methods). We detected 54,132 expressed genes in at least one of the 11 samples (log2 (RPKM) ≥ -2), representing 81.8% of all 66,210 annotated soybean genes [4]. The number of detected genes among tissues varied substantially, ranging between 36,802 and 46,563 (Figure 1a), with more genes detected in active tissues, consistent with the results in rice [18]. In comparison to the recently detected 52,947 expressed genes in soybean [5], 47,162 of them were identified in our dataset and 5,805 genes were not included (Figure 1b), while our data detected additionally 6,970 expressed genes that are not present among 52,947 genes (Figure 1b). Among previously defined 46,430 high-confidence genes [4], 42,713 (92%) genes were transcriptionally active in our dataset, while 3,717 (8%) genes were undetected (Figure 1b). Conversely, our data also detected additional 11,419 genes previously defined as low-confidence genes [4], including 5,284 genes from 12,673 recently designated as pseudogenes [5] (Figure 1c), suggesting that ultra-high throughput sequencing coupling with multiple tissues is capable to identify more low level or tissue preferentially expressed genes. Altogether, integration of this study and previous data suggest that a total of 61,849 genes (nearly the annotated genes in soybean genome) are transcribed, significantly improving the soybean transcriptome annotations.

### Analysis of the duplicated genes caused by latest WGD

Gene duplication is one of the most important mechanisms for understanding the evolutionary novelties, while divergence of the duplicated gene expression is highly correlated with their functional divergence [33]. Soybean has served as an attractive model plant to study this aspect due to the occurrence of two recent WGDs. Based on the annotated genes in the soybean genome, we identified 2,713 and 37,746 duplicate genes (2–6 copies) caused by TD and WGD, respectively. These genes were further divided into five types regarding copies of 2 (9728/WGD and 574/TD), 3 (283/WGD and 90/TD), 4 (145/WGD and 13/TD), 5 (22/WGD and 8/TD) and 6 or more (7/WGD and 1/TD) (Figure 1d). Our 11 samples detected 35,569 (94.23%) and 2,139 (78.84%) duplicated genes by WGD and TD, indicating that the vast majority of the existing duplicated genes by WGD are expressed. To further investigate the expression difference among tested tissues between duplicated genes, we focused on the 9,728 pairs of paralogs from WGD. Our results showed that 8,768 pairs had detectable expression for both copies, 701 pairs showed expression in one of the copies, while 259 pairs has no detectable expression in either copy (Figure 1e). Among the 8,768 two-copy expressed genes (unless otherwise noted, paralogs mentioned in following text refer to the pairs), *t*-test statistical analysis showed that 4,407 of them (50.26%) showed significant expression difference between the two paralogs (p < 0.05) (Figure 1f and in Additional file 2: Table S3), indicative of regulatory subfunctionalization and/or neofunctionalization, whereas the other 4,361 paralogs (49.74%) had no significant difference each other (p < 0.05) (Figure 1f, in Additional file 2: Table S3), suggesting functional conservation and possible redundancy between two paralogs. In addition, the lack of expression for one copy of the 701 pairs with single copy expression suggested that they are likely candidates for regulatory nonfunctionalization, although some of them are possibly additional examples of sub/neofunctionalization as they might be expressed in other tissues not sampled here or under different growth conditions. Similar trends were also found for 574 TD genes (Figure 1e).

### Transcriptome analysis identifies ~6,718 high-confidence NTRs in soybean

RNA-Seq has been widely applied to identify NTRs in *S. cerevisiae* and *S. pombe*[13,34], *Arabidopsis*[35], rice [19,22], mouse [36] and human [37]. Our transcriptome data showed that a large number of reads mapped to annotated intergenic regions, suggesting that they are potential NTRs. We therefore assembled the mapped reads to obtain 19,752 NTRs. By placing stringent requirements for the size >150 bp and read number >10, as well as being detected in at least two samples, we obtained a total of 6,718 high-confidence NTRs with a mean length of ~372 bp, 2,265 of which were reported previously [5].

**Figure 2:**
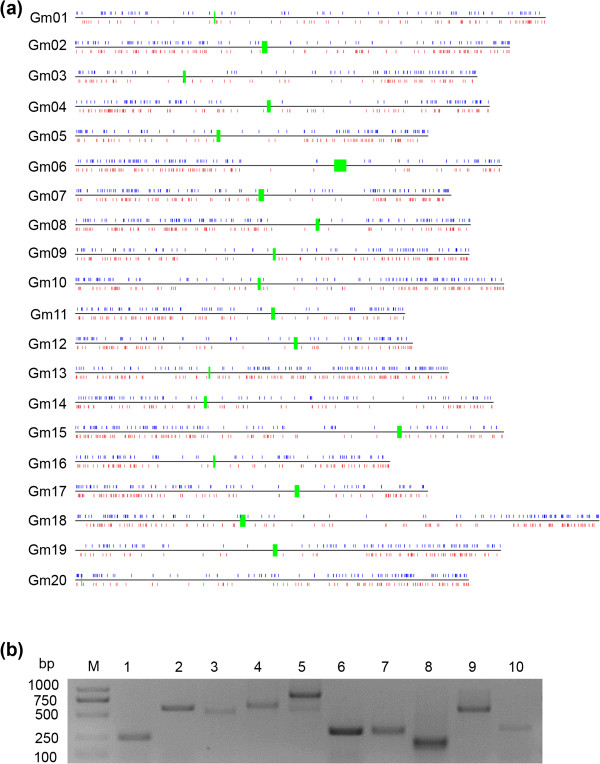
**Discovery of NTRs. (a)** Genome-wide distribution of nTUs plotted at their mapped chromosomal positions. Green shows centromere region, while blue and red show nTUs on the positive and negative strand, respectively. **(b)** Validation of the 10 randomly selected nTUs by RT-PCR.

It has been reported that NTRs are either likely novel genes or represent extension of nearby annotated transcripts, probably constituting novel exons. To test the second possibility, we searched for annotated genes within a short distance (405 bp) from the putative NTRs in the same orientation for transcription, and found that 1,509 of 6,718 NTRs were detected to extend the 5’UTR of annotated genes by in-house script (in Additional file 2: Table S4). Further analyses of these novel and extended UTRs should be helpful to the identification of additional regulatory elements. Besides the 1,509 extended genes, the other 5,209 NTRs were assembled into 4,949 novel transcript units (nTUs), evenly distributed among 20 chromosomes, but enriched in chromosome arms (Figure 2a and in Additional file 2: Table S5). Moreover, 10 randomly selected NTRs were verified as true transcripts by RT-PCR (Figure 2b), further supporting the reliability of the identified NTRs.

Among 4,949 nTUs, 2,326 (47%) were supported by the annotated 1,532 soybean ESTs in National Center for Biotechnology Information (NCBI) (in Additional file 2: Table S6), but not currently annotated in the *G. max* genome. 698 of the other 2,623 (53%) nTUs were found to have homologs from other species (in Additional file 2: Table S7), suggesting that they might be conserved genes. Only 47 nTUs were located in the transposable element (TE) regions, indicating TE activity (in Additional file 2: Table S8). To identify potential non-coding RNAs from the 2,623 nTUs, we performed a BLASTN alignment using nTUs against Rfam, and found that 40 nTUs were annotated non-coding RNA as either tRNA, rRNA, snoRNA or miRNA (in Additional file 2: Table S9). For example, *XLOC_015015* was annotated as *miR159*, suggesting that some of the novel nTUs are functional as non-coding RNAs. The nature of the remaining nTUs needs to be further investigated.

We then analyzed the spatial-temporal distribution of 4,949 nTUs in the 11 tissues (in Additional file 1: Figure S3), and found that 1,393 of them showed constitutive expression, while 3,556 were tissue preferentially expressed. Interestingly, the current soybean genome only annotates one *CLAVATA1A* (*CLV1A*) gene as the ortholog of the *Arabidopsis CLV1* gene regulating meristem sizes [38], while the identified *XLOC_047893* nTU is a paralog of *CLV1A* in soybean. Both genes showed specific expression in SAM17D and SAM38D, suggesting a redundant function of *CLV1A* and *XLOC_047893* for regulating SAM in soybean.

### Alternative spliced transcripts and their differential expression

AS is one of major contributors for generation of proteomic and functional complexity in higher organisms [16], but at present little is known about AS events in soybean. Among the previously annotated 66,210 soybean genes, 52,460 genes have at least two exons [4]. We identified a total of 12,810 AS events covering 7,084 genes (including 504 paralogs) in the 11 samples (in Additional file 2: Table S10), indicating that ~15.9% of multiple-exon genes have AS patterns. This is significantly lower than 48% observed in either *Arabidopsis* or rice [19,20,22]. A possible reason is that soybean has experienced two recent genome duplications, which resulted in many retained duplicated genes that are also a major source of proteomic and functional complexity [39].

**Figure 3:**
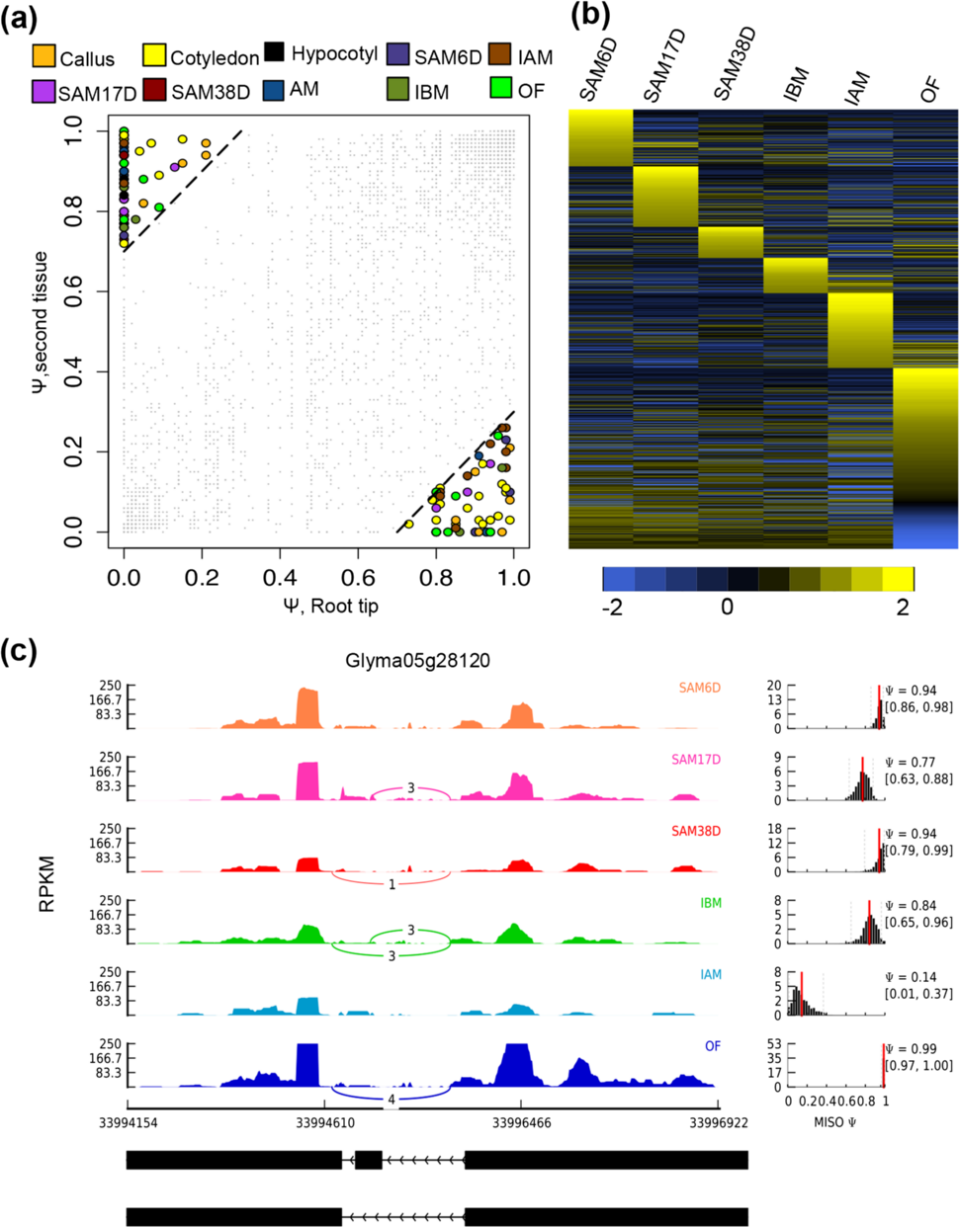
**Tissue-bias and developmentally regulated splicing events. (a)** Scatter plot shows Y values of skipped exons, and switch score was determined on the basis of comparison of root tip (x-axis) with a second tissue (y-axis). Exons with a switch score 0.7 were shown as filled symbols; others were shown as small grey dots. **(b)** Heatmap showed the regulated alternative splicing events during SAM development. The scale bar indicates Z-scores of Ψ. **(c)** Alternative splicing in *Gm05g28120* was regulated during SAM development.

We also summarized the possible existence of 11 AS types in soybean, including four common types of intron retention (32.2%), ES (26.3%), A3SS (20.8%), A5SS (11.2%) (Table 1). Unlike the major type of ES in animals [15,16], intron retention was the major type of AS in soybean, consistent with the observations in *Arabidopsis*, rice, maize and soybean [19,20,23,40]. Our result and those from others suggest that the mechanism for regulation of IR in plants is conserved. The higher proportion of ES events (26.3%) in soybean is also in agreement with that in rice and maize [19,23], but significantly higher than that in *Arabidopsis*. ES has been reported to be involved in regulating tissue-specific functions [16]. To investigate the tissue-specific expression of AS, we performed a MISO program analysis [41] to identify 202 tissue-bias exon skipped events, including 2 paralogs (in Additional file 2: Table S11). Most of them encode enzymes and transcription factors that are enriched for protein degradation, RNA regulation, signaling and transport. We also found that several exons are recognized predominantly as exons in one tissue and also as introns in another tissue. For example, as shown in Figure 3a, the 7th exon of *Gm15g15960*, showed Ψ with 88% in root tip and 6% in cotyledon (in Additional file 2: Table S11), suggesting divergent functions between root tip and cotyledon. In addition, 1,834 AS events changed greatly during SAM and flower development (Figure 3b and in Additional file 2: Table S12), GO analysis indicated that many genes encoding proteins participate in the reproductive development process. In addition to known flowering genes exhibiting AS changes, many uncharacterized genes were also observed to have significant AS changes, as exemplified by *Gm05g28120*, a gene with three sets of exons with mutually exclusive expression patterns (Figure 3c).

**Table 1:** Classification of AS in soybean

**Type of events**	**Diagram**	**Detected**	**Annotated**	**Novel**
IR		3493 (32.2%)	1814	1679
ES		2862 (26.3%)	548	2314
A3SS		2259 (20.8%)	1458	801
A5SS		1212 (11.2%)	706	506
A5SS or A3SS		359 (3.3%)	144	215
IR1 + IR2		295 (2.7%)	89	206
A5SS or A3SS		180 (1.7%)	90	90
IR1 or IR2		140 (1.3%)	46	94
MXE		44 (0.4%)	8	36
A5SS + A3SS +ES		11 (0.1%)	3	8
A5SS + A3SS +ES1 + ES2		5 (0.054%)	5	0
Total events		10860 (100%)	4911	5949

▬, Constitutive; ▭, Alternative.

### Comparison of tissue transcriptomes indicative of conservation and divergence

**Figure 4:**
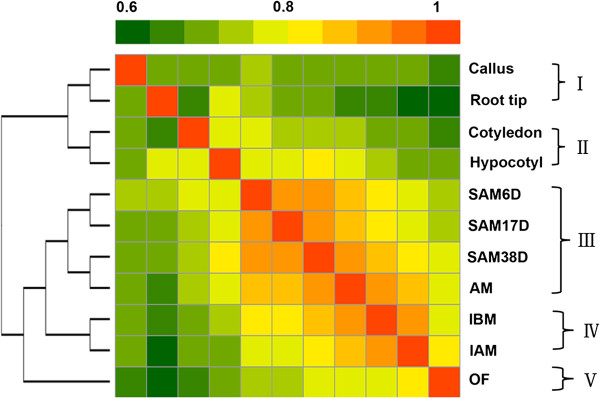
**Comparison of the transcriptome relationships among the examined tissues.** The correlation matrix was performed by comparing the values of the whole transcriptome (54,132 genes) in 11 tissues, using the log_2_ transformed gene expression value (RPKM) and Pearson’s distance as the metric. Correlation analysis was performed using R software.

To investigate the similarity of the 11 tissues, we compared their transcriptomes to generate a heatmap on the basis of Pearson correlation coefficients between any two of the transcriptomes (Figure 4). The lowest coefficient value of 0.62 was between root tip and OF, whereas the highest value 0.92 was between AM and SAM38D (in Additional file 2: Table S13). We further used hierarchical clustering (HCL) to divide the 11 samples into four groups: (I) root tip and callus; (II) cotyledon and hypocotyl; (III) SAM6D, SAM17D, SAM38D and AM; (IV) IBM and IAM; (V) OF (Figure 4). The similarity of root tip and callus in Group I is consistent with a previous discovery in *Arabidopsis* that callus, even when derived from aerial organs, resembles the development of root apical meristem in terms of specific gene expression profiles [42]. A recent study further solidified this similarity because the process of leaf-to-callus transition involves epigenetic activation of the root preferential gene expression [43]. Observation of the similar transcriptomes in soybean suggests that the molecular mechanism to determinate cell fate for callus formation could be conserved in plants. Cotyledon and hypocotyl were clustered other, suggesting they are more similar as compared with other tissues (Figure 4). In comparison to the four tissues above, the other seven samples were grouped into one clade, as supported by close Pearson’s correlation coefficient values, especially for AM with either SAM38D or IBM. Additional comparison among the three tissues identified 1,884 overlapping genes (in Additional file 1: Figure S4), which are mainly involved in the reproductive cellular processes, such as floral organ determination and stamen development (in Additional file 2: Table S14), indicating that AM at this developmental state shares some common features between shoot AM and floral meristem. Taken together, these results suggest that organ identity and cell fate determination are highly regulated by the temporal and spatial expression of genes.

### Identification of tissue-preferentially expressed genes

**Figure 5:**
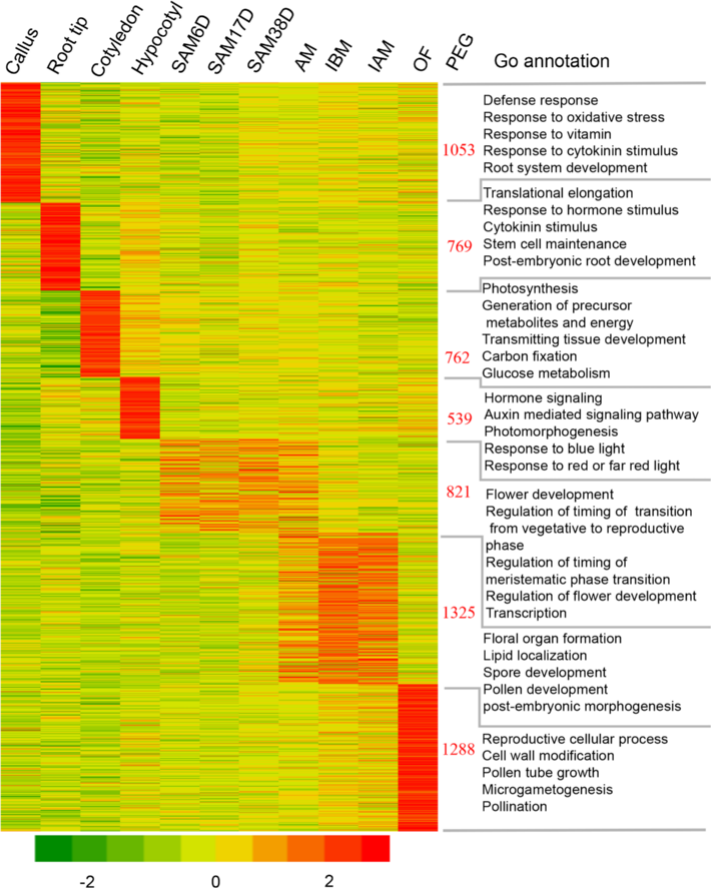
**Tissue preferentially expressed genes in 11 examined tissues of soybean.** The heatmap visualized the self-division of preferentially expressed genes in individual or grouped tissues. Color gradient illustrated the Z-scores of the gene expression values by calculating as the mean-centered log_2_ (RPKM) values divided by the standard deviation for each gene, separately. Right to the heatmap showed the number of preferential genes and selected significantly enriched GO terms (P < 0.001).

Characterization of tissue or cell-specific genes provides a foundation for unraveling their molecular mechanisms. Previous studies in multiple plants demonstrated that each organ or tissue has its specific transcripts [18,21,44], including soybean [5]. These genes expressed more highly in one tissue (or a closely related set of tissues) than all other tissues examined are referred to as preferentially expressed genes (PEGs). To investigate the tissue PEGs, we first compared the transcriptomes among 11 tissues and found 6,557 tissue PEGs (Figure 5). Among these genes, root tips had 769 PEGs, including 65 paralogs; GO annotation showed that they were related to translational elongation, hormone signaling, cytokinin stimulus, stem cell maintenance and post-embryonic root development (Figure 5, in Additional file 2: Tables S15 and S16). In *Arabidopsis*, *PIN2* is specifically required for auxin transport during root development [45]. Two paralogs similar to *PIN2* were found in soybean and showed similarly specific expression in root tips, suggesting that they have possibly redundant function in root development similar to that of *PIN2* in *Arabidopsis*. In contrast, 1,053 PEGs identified in callus, including 102 transcription factors and 48 paralogs, were mainly involved in biotic and abiotic responses, such as defense, oxidative stress, vitamin, inorganic substance and cytokinin stimulus (Figure 5, in Additional file 2: Tables S17 and S18). The 762 cotyledon PEGs (49 paralogs) were enriched in photosynthesis, energy, transmitting tissue development and glucose metabolism (Figure 5, in Additional file 2: Tables S19 and S20). Auxin is a crucial regulator of cotyledon development [46]. We detected several other auxin-related genes, including the pair of *Gm09g38700* and *Glyma18g47630* paralogs that are homologs of *Arabidopsis PIN-FORMED 5 (PIN5)*, which is required for auxin homoeostasis and gametophyte development [47,48]. However, both genes were found with highest expression in cotyledon, but nearly undetectable in reproductive tissues, suggesting *PIN5* may have a divergent role in soybean. In contrast, the 539 hypocotyl PEGs (27 paralogs) were enriched for an auxin-mediated signaling pathway, and/or photo morphogenesis (Figure 5, in Additional file 2: Tables S21 and S22), including homologs of the *Arabidopsis NON-PHOTOTROPIC HYPOCOTYL 3 (NPH3)* gene [49].

As shown in Figure 4, AM was highly similar to both SAM38D and IBM, pairwise comparison would probably miss many genes active in meristems. To identify PEGs in these meristems (but not specifically in one of them), we grouped similar meristems together and detected 821 genes (20 paralogs). GO annotation indicated that the most enriched categories were associated with flower development and regulation, floral transition from vegetative to reproductive phase, or meristematic phase transition and transcription regulation (Figure 5, in Additional file 2: Tables S23 and S24), which is in good agreement with previous reports in soybean [50,51]. For instance, the PEGs included several homologs of *SHORT VEGETATIVE PHASE* (*SVP*) that specify the reproductive organ identity and control flowering time in *Arabidopsis* and rice [52,53] and genes involvement in *WUSCHEL* (*WUS*) regulatory network essential for SAM maintenance [54]. We also found homologs (*Gm14g15820* and *Gm7g30920*) of genes for auxin synthesis and response, such as *YUCCA4*, in accordance with the fact that the *Arabidopsis YUCCA4* expression is restricted to the SAM and flower meristems or young floral primordia [55], as well as 20 genes related to auxin-responsive genes regulating SAM development [56]. These good agreements between our GO enrichment results and known functions in meristem suggested the reliability of the collected samples for SAM and conservation of molecular mechanisms for controlling SAM between *Arabidopsis* and soybean.

Accordingly, AM, IBM and IAM together had 1,325 PEGs (60 paralogs) (Figure 5, in Additional file 2: Table S25), which were mainly involved in reproductive processes, such as floral organ determination and development, stamen development, tapetal layer development, pollen development (Figure 5). For instance, in addition to the identification of several flower organ identity genes from ABC model (in Additional file 2: Table S26), we also found genes specifically for meiosis, such as *MS5* (*Gm08g47070* and *Gm18g38060*) and *MMD1* (*Gm14g39310* and *Gm02g41020*) [57]*.* Unlike the expression of *Arabidopsis MS5* and *MMD1* genes restricted in meiocytes, the soybean homologs showed high expression in AM, suggesting a possible unknown function in soybean. Interestingly, the *Arabidopsis DREB1B* is one of the critical regulators for cold responses, and is also widely expressed [58], whereas the soybean homologs (*Gm11g19340* and *Gm12g09130*) showed special expression in AM, IBM and IAM, but not in other vegetative tissues, suggesting it might have gained novel functions in reproductive development in soybean. In addition, one homolog of *DREB1A* (*Gm17g14110*) was also identified, consistent with a recent novel discovery that the *Arabidopsis DREB1A* gene is important for flower development especially under unfavorable conditions [59].

Finally, open flower had 1,288 PEGs (78 paralogs) enriched for reproductive cellular process, cell wall modification, pollen tube growth, pollination and signal transduction (Figure 5, in Additional file 2: Tables S27 and S28). Particularly, at least 50 genes (most in two copies) encoded signal transduction proteins for interaction between the pollen and ovary, such as *SNAP receptor 124*, *leucine-rich repeat protein kinase*, *ROP BINDING PROTEIN KINASES 1*, *calcium-dependent protein kinase 24*[60,61].

### Dynamic reprograming of soybean SAM transcriptome

**Figure 6:**
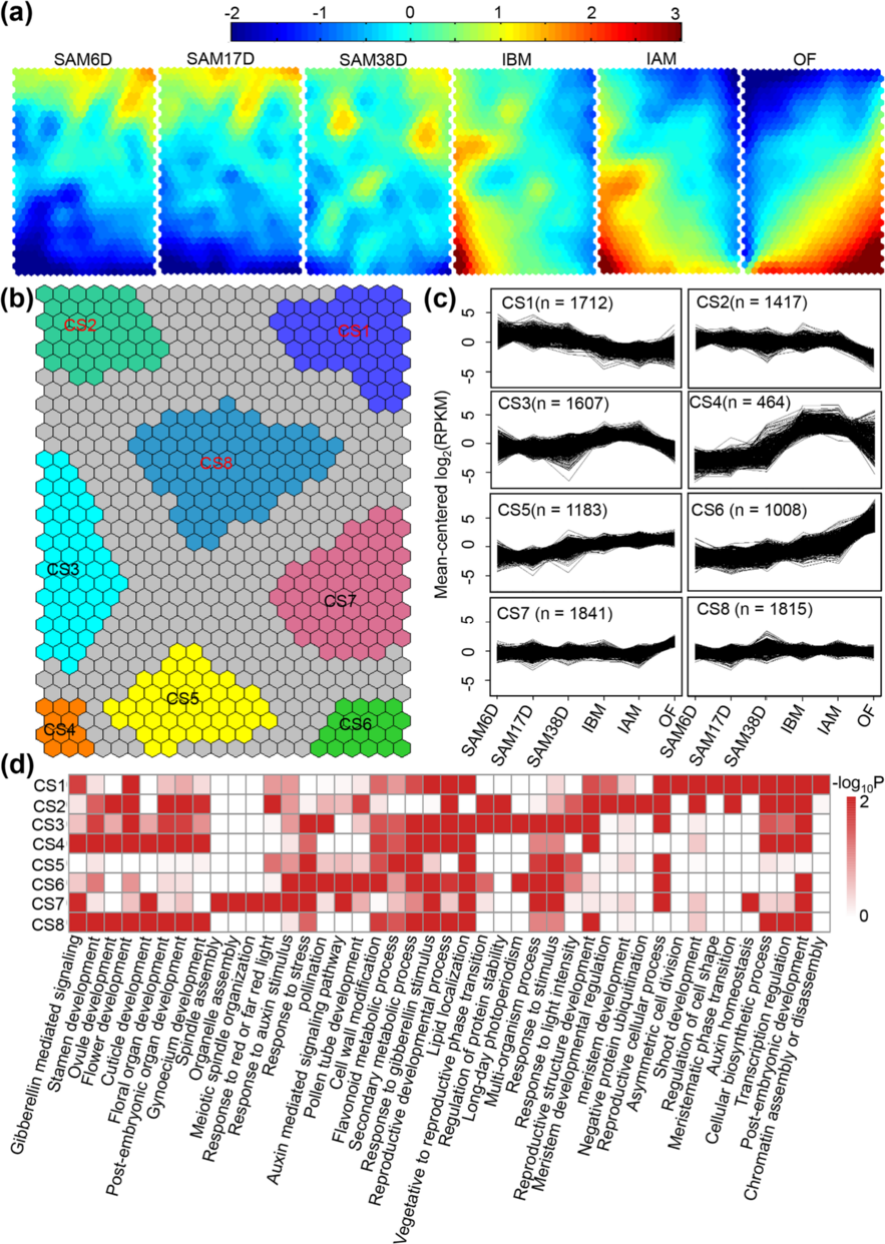
**Expression patterns of genes regulated during soybean SAM development. (a)** Component planes of a SOM fitted to the developmental stage data set. Each component plane visualizes mean-centered gene expression (log_2_-scale) in one stage as a color gradient from blue to red, indicating low and high expression, respectively. **(b)** Eight regions (CS1-CS8) of the SOM were robustly clustered together. **(c)** Mean-centered log_2_-expression values of genes corresponding to eight of the clusters in **b** were plotted for the 50% of best-fitting genes. **(d)** Functional category enrichment by AgriGO among the eight major clusters.

Comparison of genes between soybean and *Arabidopsis* provides clues regarding conservation of critical genes for SAM development. To obtain clearer transcriptome changes during SAM development, we mainly focus on 22,571 DEGs during soybean SAM and flower development (in Additional file 2: Table S29). Verification of expression of randomly selected 20 genes in SAM by qRT-PCR, showed a high correlation (R^2^ = 0.93) with RNA-seq (in Additional file 2: Table S30), supporting the reliability of our dataset. We then applied self-organizing maps (SOMs) [62] to seek shared patterns of DEGs in relation to the developmental stage (Figure 6a), and subsequently identified eight regions (CS1-CS8) on the basis of similarly shared patterns (Figure 6b and in Additional file 2: Table S31). Among them, genes in CS1 were expressed above an average level in early stage of SAMs, but below the average level in later stage of IBM, IAM and OF (Figure 6c), indicating they are important for early SAM development, but not afterwards. GO enrichment analysis showed that those genes mainly participate in chromatin assembly and disassembly, regulation of transcription, regulation of timing of meristematic phase transition, asymmetric cell division and auxin homeostasis (Figure 6d and in Additional file 2: Table S32), suggesting a vital role of transcription regulation for early SAM development. Genes in CS2 and CS7 showed stable expression in the five early reproductive tissues excluding OF, but exhibited sharply decreased and increased expression in OF, respectively (Figure 6c). This indicates that the CS2 genes have roles in early flower development, but are not as important for the later stage. Genes with such expression mainly participate in meristem development, reproductive structure development, and transcription regulation, as well as the negative regulation of protein ubiquitination (Figure 6d and in Additional file 2: Table S32). In contrast, the CS7 genes are more active in the later stage of flower development. Those genes are involved in responses to stimulus, auxin signaling, lipid localization and spindle organization (Figure 6d and in Additional file 2: Table S32). Genes in CS3 and CS4 showed similar expression patterns with an increase from SAM38D to IAM and then a decrease in OF, but the increased levels are much higher in CS3 than those in CS4 (Figure 6c), suggesting that those genes could be important for post-meiotic flower development. Indeed, gene involvement in reproductive development was enriched by GO analysis (Figure 6d and in Additional file 2: Table S32), including genes from the ABC model and those required for anther or pollen development. Expression levels of genes in CS5 and CS6 gradually increased with the developmental stage in SAM (SAM6D, SAM17D, SAM38D) and reproductive (IBM, IAM, OF) tissues, with CS6 genes showing higher expression levels in OF than genes in CS5 (Figure 6c). This indicates that the genes with elevated expression in OF from CS6 are more active for later reproductive development processes, such as pollen tube development and pollination, as supported by GO enrichment analysis (Figure 6d and in Additional file 2: Table S32). Genes in CS8 were constitutively expressed in six tissues, and part of them showed fluctuating expressions in SAM38D (Figure 6c). Those genes are important not only for basic cellular development, but also for meristem and flower development (Figure 6d and in Additional file 2: Table S32). Together, further functional studies of genes from different clusters could contribute to a better understanding of the biological implications of them during SAM and flower development in soybean.

### Distinct expression of transcription factors in SAM

**Figure 7:**
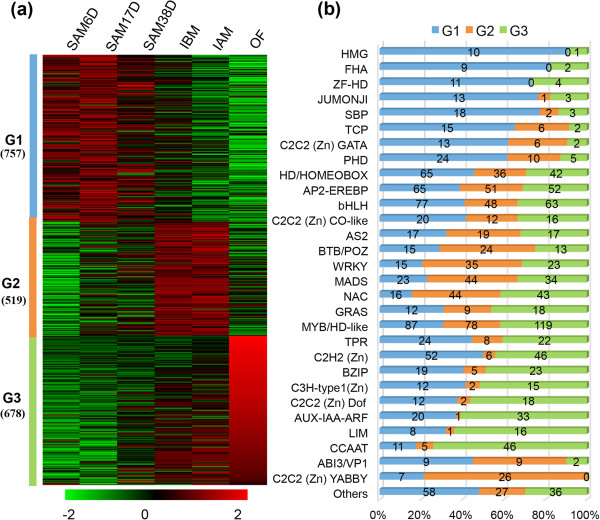
**Dynamics of transcription factor profiles. (a)** Dendrogram of the transcription factors. 1,954 differentially expressed transcription factors from the six tissues (RPKM > 1 in at least one segment) clustered into three lineages (G1, G2 and G3) using the K means-clustering method. **(b)** Distribution of transcription factor families among G1, G2 and G3.

Identification of the dynamically accumulated TFs during soybean SAM and flower development is an initial step in understanding the underlying regulatory networks. Current soybean genome is annotated with 5,671 TF genes, which are classified into 63 different families [4]. We detected a total of 4,806 TF genes (642 paralogs) with expression in at least one of six samples (SAM6D, SAM17D, SAM38D, IBM, IAM, OF). 1,954 of them (141 paralogs) were differentially expressed (GFOLD >1 or GFOLD < -1; RPKM >1) (Figure 7a and in Additional file 2: Table S33), uncovering nearly all families. We then classified the 1,954 TF genes into three clusters according to distinct expression patterns (Figure 7a). 39.8%, 29.6% and 30.6% of these TF genes were expressed at the highest levels in SAMs (designated as G1), IBM and IAM (designated as G2), or OF (designated as G3), respectively (Figure 7b).

**Figure 8:**
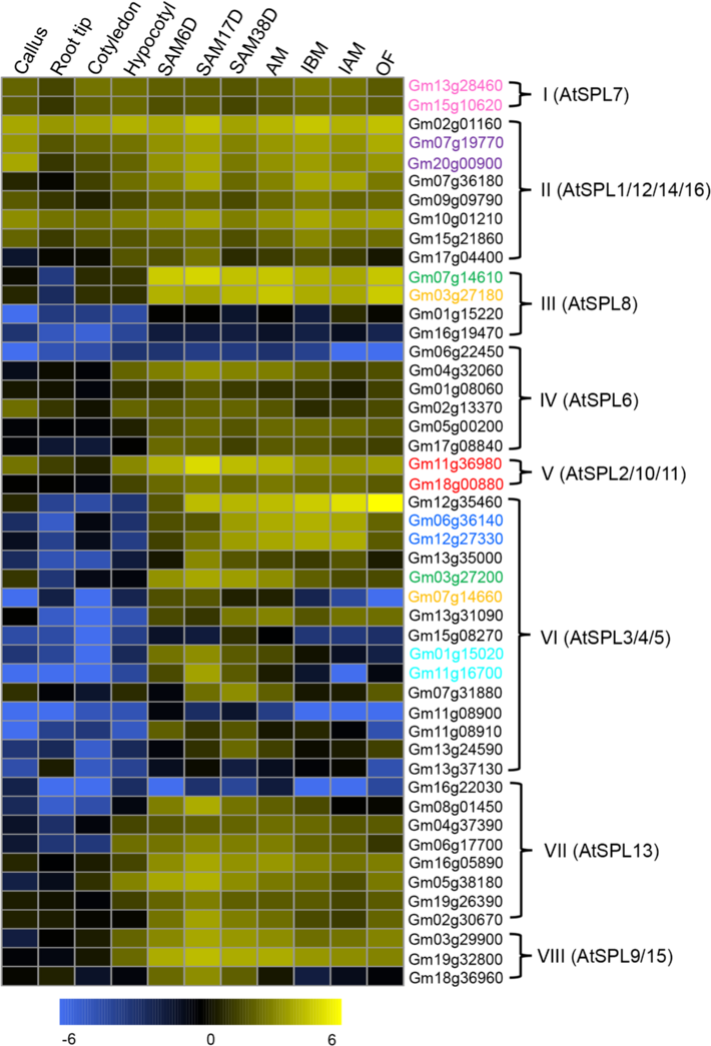
**Heatmap analysis of the soybean *SPL *transcription factor family in 11 tissues.** The I-VIII clades were divided by phylogenetic analysis in Additional file 1: Figure S8. Only *Arabidopsis* genes were list in each clade as reference. Name of genes marked in same color are a pair of paralogs.

Further classification of family-preferential expression showed that G1 mainly includes families of HMG, FHA, ZF-HD, SBP, TCP, C2C2 (Zn) GATA and PHD (Figure 7b), indicating that early SAM development largely requires those transcription factor families. For example, *SQUAMOSA-PROMOTER BINDING PROTEIN-LIKE (SPL)* proteins are a family of plant-specific TFs having a conserved SBP (*SQUAMOSA* promoter binding protein) domain, and play multiple roles in plant growth and development [63]. 16 and 48 *SPLs* are found in *Arabidopsis* and soybean, respectively [64], and were divided into eight clades (in Additional file 1: Figure S5 and in Additional file 2: Tables S34-S35). 23 *SPLs* from 7 clades were differentially expressed during soybean SAM and flower development. Available data from *Arabidopsis*, rice and tomato support the idea that the function of genes from some different clades might still be conserved, but genes from other clades might have diverged [63-65]. For instance, 10 of 16 *Arabidopsis SPLs* (*SPL2-6, SPL9-11, SPL13,* and *SPL15*) from 5 clades are *miR156/157* targets [66], and play a similar role in phase transition [65], whereas the clade I-, II- and III-containing genes lack *miR156* and *miR157*-binding sites. The clade I contains only *SPL7* with ubiquitous expression and distinct function in regulating copper homeostasis [67]. Consistently, two soybean *SPL7* paralogs are also widely expressed with similar patterns, suggesting a conserved role in soybean (Figure 8). Clade II has four members of *SPL1, SPL12, SPL14* and *SPL16* with wide expression in *Arabidopsis* (Figure 8), but only *AtSPL14* has been shown to participate in vegetative to reproductive transition [63]. This clade includes eight members in soybean. Seven of them showed similar expression patterns to that of *Arabidopsis* (Figure 8 and in Additional file 2: Table S36), but only *Gm17g04400* was differentially expressed in SAM, suggesting a function different from that of its counterpart. The clade III has only *AtSPL8* with a function in root growth and microsporogenesis [68]. Four soybean genes were found in this clade, and two of them had no detectable expression, suggesting possible non-functionalization. By contrast, the other two genes were specifically expressed in SAM and reproductive tissues. However, unlike *AtSPL8* being functional in roots, the soybean homologs were not expressed in roots, resembling that of tomato *SPL8* homologs [64]. The clade IV contains *AtSPL6* with constitutive expression and unknown function*.* However, a *Physcomitrella patens* homolog has been reported to repress reproductive development [69], somehow similar to *AtSPL14*[70]. This clade had six soybean homologs; except for the undetectable expression of *Gm06g22450*, the other five genes were highly expressed in SAM and reproductive tissues. In comparison to the other 7 clades having more genes from soybean than *Arabidopsis*, the clade V has three Arabidopsis genes, but only two paralogs from soybean (Figure 8). Therefore, it would be interesting to investigate possible reasons to cause gene lost on soybean in this clade.

The most extensively characterized function of *SPLs* is promotion of the transition from vegetative and reproductive growth, and particularly for *SPL3-5* in clade VI of *Arabidopsis*[71]. Remarkably, this clade contains 15 *SPLs* from soybean, 14 of which showed high expression in SAM (Figure 8) and were nearly undetectable in other tissues, suggesting the conservation of molecular mechanism in regulation of the transition from vegetative and reproductive growth between *Arabidopsis* and soybean. The last two clades of VII and VIII include *AtSPL13* and *AtSPL9/15*, respectively (in Additional file 1: Figure S5). *AtSPL13* has been implicated in leaf development, while the *AtSPL9* and *AtSPL15* play a partially redundant role in phase transition [72,73]. The seven and four *SPL* genes in soybean in clade VII and VIII had very similar gene expression patterns in SAM and floral tissues, consistent with the functions of the *Arabidopsis* homologs. Together, 7 paralogs pairs were included in *SPL* family (Figure 8). Comparison of expression patterns suggests that the paralogs in a pair might have undergone sub-functionalization, further supporting the idea that sub-functionalization might be predominant event for duplicated gene after WGD in soybean.

Different from G1, G2 mainly contained MADS, AS, BTB/POZ, WRKY, C2C2 (Zn) YABBY (Figure 7b). It has been reported that MADS-box gene family is not only key repressors or activators for flowering transition, but also as master regulators of reproductive organ identities [74]. Our data detected 101 MADS-box genes during flower development (Figure 7b), such as *Gm01g08150, Gm04g42420, Gm08g12730* and *Gm08g27670*, which are homologs of *AP1, PI, AG* and *SEP2*, respectively, consistent with their known function in floral organ identity. Therefore, the functions of the MADS-box gene family for regulation of flower development are likely conserved between soybean and *Arabidopsis*. In contrast, BZIP, C3H-type1 (Zn), C2H2 (Zn) Dof, AUX-IAA-ARF, LIM and CCAAT gene families were preferentially expressed in OF (Figure 7b). Many studies showed that auxin-dependent transcriptional regulation requires the auxin/indole-3-acetic acid (Aux/IAA) and auxin response factor (ARF) families of TFs [75] and formation of Aux/IAA-ARFs heterodimers repress auxin signaling [75]. In addition to the known role of auxin in *Arabidopsis* pollen development, pollination and fertilization also seem to need increased auxin levels [76]. Indeed, we detected 33 differentially expressed members in OF, suggesting Aux/IAA-ARF regulatory pathway for later reproductive development is also conserved. However, the function of other enriched TFs in OF is still largely unknown.

## Conclusions

The paleopolyploidy and rapid divergence of the soybean genome makes it an ideal genome for evolutionary analyses. However, the present soybean genome annotation and gene expression message are incomplete. This study presents the overall transcriptional landscape and provides extensive evidence that transcriptional regulation in soybean is vastly more complex than previously expected. The data significantly improves annotation of the soybean genes predicted in genome, as well as provides essential sources for studying the expression level between duplicated genes by latest WGD and functional genome in soybean.

## Methods

### Plant material and growth condition

Soybean (Glycine max) plant materials used here were from the HX3 cultivar. Three-day after germination and older seedlings were generated on a quartz sand culture under a 14 h/10 h light/dark regime at 28°C (in light)/25°C (in dark) with 70% relative humidity and used to obtain root tips of 0.2-0.3 cm in length. Similarly prepared four-day seedlings were used to collect cotyledons and hypocotyls. SAMs (shoot apical meristems) at 6, 17 and 38 days after germination were collected from soil grown plants, using tweezers and a dissecting needle. Axillary meristems were collected under the second or third internode of shoot apex of soil grown plants after 38-day germination. Each meristem RNA-seq sample included materials from ~1000 plants. For inflorescences pre- or post-meiotic stage, we defined an appropriate size of inflorescence by analyzing tetrad and chromosome spread, and then dissected the inflorescences from 45-day soil-grown plants under microscopy, and separated open flowers from unopened buds. Callus induction was carried out using the cotyledonary-node method as described previously [77] with minor modification [78]. All samples were taken at room temperature 25°C and quickly placed in liquid nitrogen.

### RNA isolation, RNA-seq library preparation and sequencing, real-time RT-PCR

RNA isolation, RNA-seq library preparation and sequencing were performed using the protocols described previously [21,28,29]. RT-PCR was carried out according to a previous procedure [21,29]. Primers used in this study were listed in Additional file 2: Table S30. Fold change for gene expression was calculated by normalizing Ct values at each developmental stage against endogenous control (Gmβ-actin: *Gm15g05570*) using the 2^-ΔΔCt^ method [79].

### Mapping of reads and calculation of gene expression level

Reads obtained by SOLiD sequencing were aligned against soybean genome assembly version 9 (Glyma1.1; http://www.phytozome.net/), using the Lifescope software package. Lifescope used a seed-and-extend approach to map reads against the reference. The normalized gene expression level was calculated as Reads Per Kilo-base of mRNA length per Millions of mapped reads (RPKM) by the GFOLD V1.0.7 software [80]. A comparison between the expression levels of genes and intergenic regions was used to find a threshold for detectable expression above background. The value of 0.25 RPKM was the threshold classifying annotated genes into two large clusters, and was defined as the threshold between “expressed” and “unexpressed”. Next, DEGs (differentially expressed genes) were defined using GFOLD diff program (GFOLD >1 or GFOLD < -1; log2 (fold change) >2 or log2 (fold change) < -2). The preferentially expressed gene for specific tissue was defined by meeting at least GFOLD >1 and RPKM > 4 in the tissue in question compared to all the other tissues.

### Identification of putative paralogs and differential expression analysis

We used the MCScanx software [81] to identify potential paralogous clusters. WGD genes and TD genes were detected with default parameters. The differential expression of paralogs was analyzed based on the Log2-normalized RPKM values across 11 samples and *t*-test to assess statistical significance.

### Correlation analysis

A correlation matrix was prepared using the R software and Pearson’s correlation coefficient as the statistical metric to compare the values of the whole transcriptome (54,132 genes) in 11 samples. Log2-normalized RPKM values from RNA-seq dataset were used to create the correlation matrix, and then R scripts were used to analyze the correlation among samples. Correlation coefficient values were converted into distance to define the height scale of the dendrogram. The heat map of the correlation was implemented by the pheatmap (Pretty Heatmaps) function in the pheatmap package (R version, 2.15, pheatmap version, 0.6.1; R Core Team, Vienna, Austria).

### Discovery of NTRs and RT-PCR validation

We used the Cufflinks software [82] to assembly transcripts using high quality mapped reads (no mismatch) from Lifescope, and obtained intergenic transcripts based on Class Code “u” comparing the annotated soybean genome ( http://www.plantgdb.org/GmGDB/), using the following criteria: (1) larger than 150 bp in size, (2) reads number > 10 and (3) supported by detection in at least two tissue samples. Based on these criteria, we obtained ~6,718 high confidence NTRs. RNA-seq reads were visualized on the soybean genome using the inGap software [83]. 10 randomly selected NTRs were verified by performing RT-PCR using specific primers designed for this study (in Additional file 2: Table S37). Additionally, the BLAST was used to identify nTUs agaist the Rfam [84,85].

### Alternative splicing analysis

We used the ASTALAVISTA software [82] to quantify the type of AS events based on the assembled transcripts by the Cufflinks software. MISO [41] and a MISO pipeline were used, respectively, to evaluate the expressed transcripts and their differential expression across the 11 samples. First, we need to generate two file libraries:annotation file of alternative splicing events and indexed alignment file. For the AS events file, we use MISO to measure differential expression by Bayesian inference. For the alignment file, the high quality-filtered reads for the different samples were aligned against soybean genome with Lifescope using the soybean genome feature file to improve the detection of splicing junctions. A combination of different cut-offs and filters were tested yielding the MISO output, culminating in the use of a Bayes factor of 0.7 as cut-off value to detect differential AS events. RNA-seq reads were visualized on the soybean genome using the sashimi plot tool with RPKM.

### Self-organizing maps

We used the SOM (Self-Organizing Maps) method [86] for both clustering and visualization of the patterns of DEGs during SAM and flower development. The SOM Toolbox for MATLAB developed by the Laboratory of Information and Computer Science at the Helsinki University of Technology was used ( http://www.cis.hut.fi/projects/somtoolbox/). One SOM was fitted to mean normalized log2-transformed (RPKM values) gene expression estimates from the data of a specific developmental stage/tissue. Regions in the SOM corresponding to characteristic and coherent expression patterns were afterward identified by k-means clustering of the SOM units (k = 8 for the developmental data set). The top half of more coherent SOM units was identified by means of silhouette coefficients resulting in the revealing clusters. Finally, we visualized prototypical gene expression patterns for each SOM region. Genes are plotted with a best-matching SOM unit within one of these regions.

### GO enrichment analysis

Gene lists were analyzed for gene ontology (GO) enrichment using the online tools AgriGO ( http://bioinfo.cau.edu.cn/agriGO/analysis.php) with Fisher’s exact test and false discovery rate (FDR) correction [87]. Transcription factor (TF) family annotations were downloaded from the soybean genome annotation, containing 5,671 TFs in 63 families for *Glycine max*[4]. The heat map of the expressed TFs was generated by a heatmap.2 function in the gplots package (R version, 2.15, R Core Team, Vienna, Austria). In addition, all gene functional descriptions were from modified MapMan annotations [88].

### Availability of supporting data

The data sets supporting the results of this article are available in the NCBI GenBank repository [ http://www.ncbi.nlm.nih.gov/bioproject/?term=PRJNA241144] and in the NCBI SRA repository [ http://www.ncbi.nlm.nih.gov/sra/?term=SRP040057].

## Abbreviations

RPKM: Reads/Kb/Million; GO: Gene ontology; RNA-Seq: RNA sequencing; SAM: Shoot apical meristem; PCR: Polymerase chain reaction; qRT-PCR: Quantitative reverse transcription polymerase chain reaction; RNA: Ribonucleic acid; TF: Transcription factor; SOM: Self-organization map; AS: Alternative splicing; NTR: Novel transcribed regions.

## Competing interests

The authors declare that they have no competing interests.

## Authors’ contributions

LW, YXW, HN and HM designed experiments. YXW, ZHC, QBM and QYZ collected tissues. LW and GFZ prepared mRNAs, cDNA libraries for SOLiD sequencing. LW conducted RT-PCR and qRT-PCR experiments. LW, CLC, HFW and JQ performed bioinformatics analysis. LW, HM and YXW wrote the paper. All authors read and approved the final manuscript.

## Supplementary Material

Additional file 1: Figure S1 Total number of reads mapped in samples and distribution of reads among soybean annotated genome. **Figure S2.** The distribution of RPKM values across 11 samples. (a) Comparison the expression level of genes (blue) and intergenic background regions (red) across 11 soybean tissues. We zoomed in the effects at expression spanning between -15 to 15 log2-transformed RPKM values. (b) The distribution of log2-transformed RPKM values across 11 samples. The vertical dashed line denotes the threshold above which the genes were determined as expressed. The log2-transformed RPKM values of genes at each sample were binned with interval size 0.1. **Figure S3.** The expression profile of the 4,949 NTUs. **Figure S4.** Comparison of the expressed genes among SAM38D, AM and IBM. **Figure S5.** Unrooted phylogenetic tree of the SBP-box family genes based on AA sequences of SBP domains. Click here for file

Additional file 2 **Table contents for S1 to S37. Table S1.** Summary of mapped reads. **Table S2.** Distribution of reads among genome. **Table S3.** The *t*-test of 8768 paralogs. **Table S4.** List of 1509 nTRs in the 5’UTR upstream of genes. **Table S5.** Distribution of 4949 nTUs on chromosomes. **Table S6.** List of 2326 nTUs and annotated genes in NCBI. **Table S7.** Go annotation of 698 nTUs. **Table S8.** The 47 nTUs in TE. **Table S9.** List of 40 nTUs. **Table S10.** List of AS events. **Table S11.** The description of 202 skipped exon gene. **Table S12.** The Psi value of 1834 transcripts. **Table S13.** Correlation matrix of the whole dataset. **Table S14.** GO annotation of overlapped genes between AM and IBM. **Table S15.** List of root tip PEGs. **Table S16.** GO annotation of root tip PEGs. **Tables S17 and S18.** List and GO annotation of callus PEGs. **Tables S19 and S20.** List and GO annotation of cotyledon PEGs. **Tables S21 and S22.** List and GO annotation of hypocotyl PEGs. **Table S23.** List of PEGs in multiple meristems. **Table S24.** GO annotation of PEGs in multiple meristems. **Table S25.** List of the PEGs in AM, IBM and IAM. **Table S26.** GO annotation of the PEGs in AM, IBM and IAM. **Table S27.** List of the OF PEGs. **Table S28.** GO annotation of the OF PEGs. **Table S29.** List of the PEGs among 6 SAM samples. **Table S30.** Correlation of mRNA-seq and qRT-PCR. **Table S31.** RPKM value and function description of eight clusters. **Table S32.** P value of each GO term. **Table S33.** Distribution of TF families among G1, G2 and G3. **Table S34.** Phylogenetic analysis of *SBP-box* family. **Table S35.** SBP-domain sequences and accession numbers of plant *SBP*s. **Table S36.** Expression of 48 soybean *SBP*s. **Table S37.** List of RT-PCR primers. Click here for file
