# Supplementary figures and images for: RNA-seq analyses of multiple meristems of soybean: novel and alternative transcripts, evolutionary and functional implications
Source: BMC Plant Biol. 2014 Jun 17;14:169. doi: 10.1186/1471-2229-14-169 (PMC4070088; doi:10.1186/1471-2229-14-169)

Figure S1

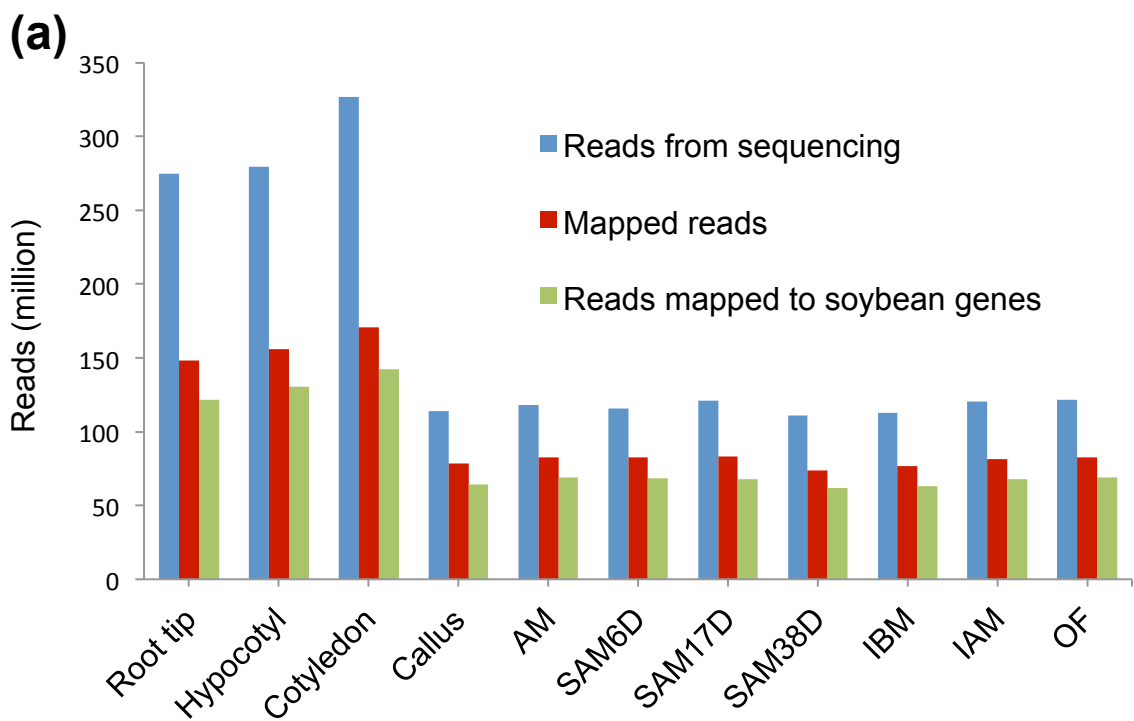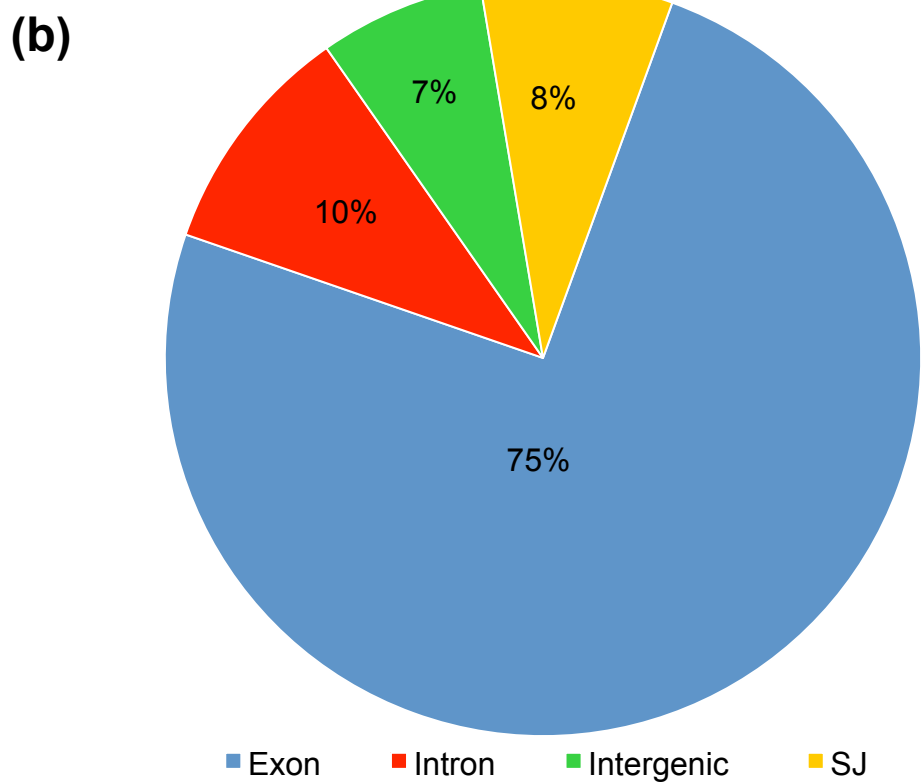

**Figure S2**

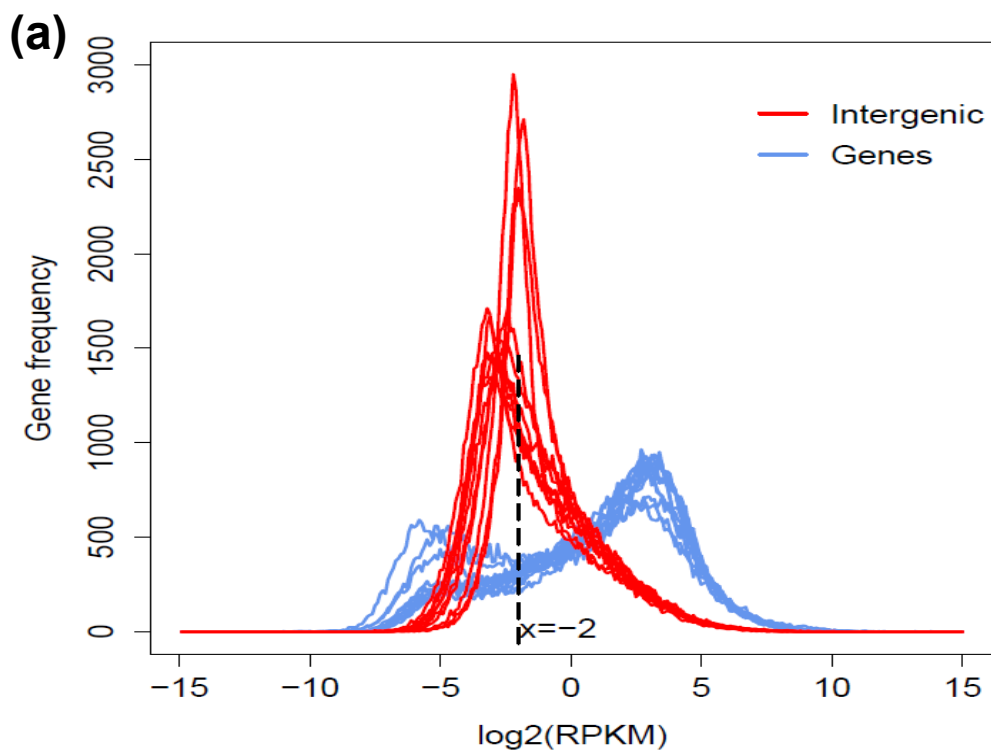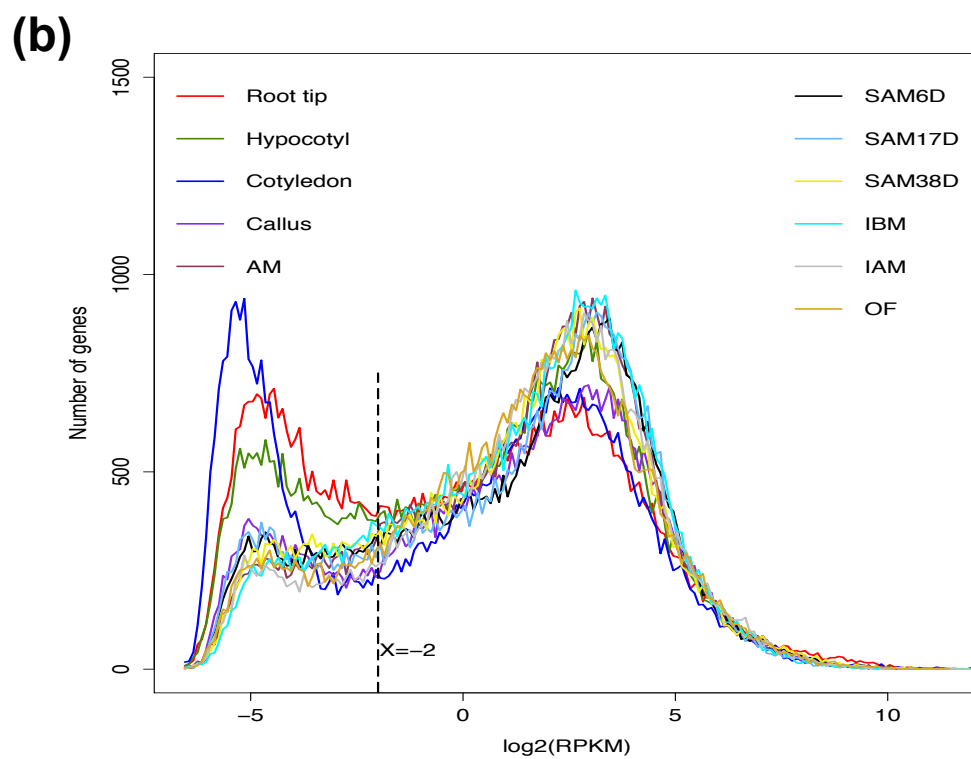

Figure S3

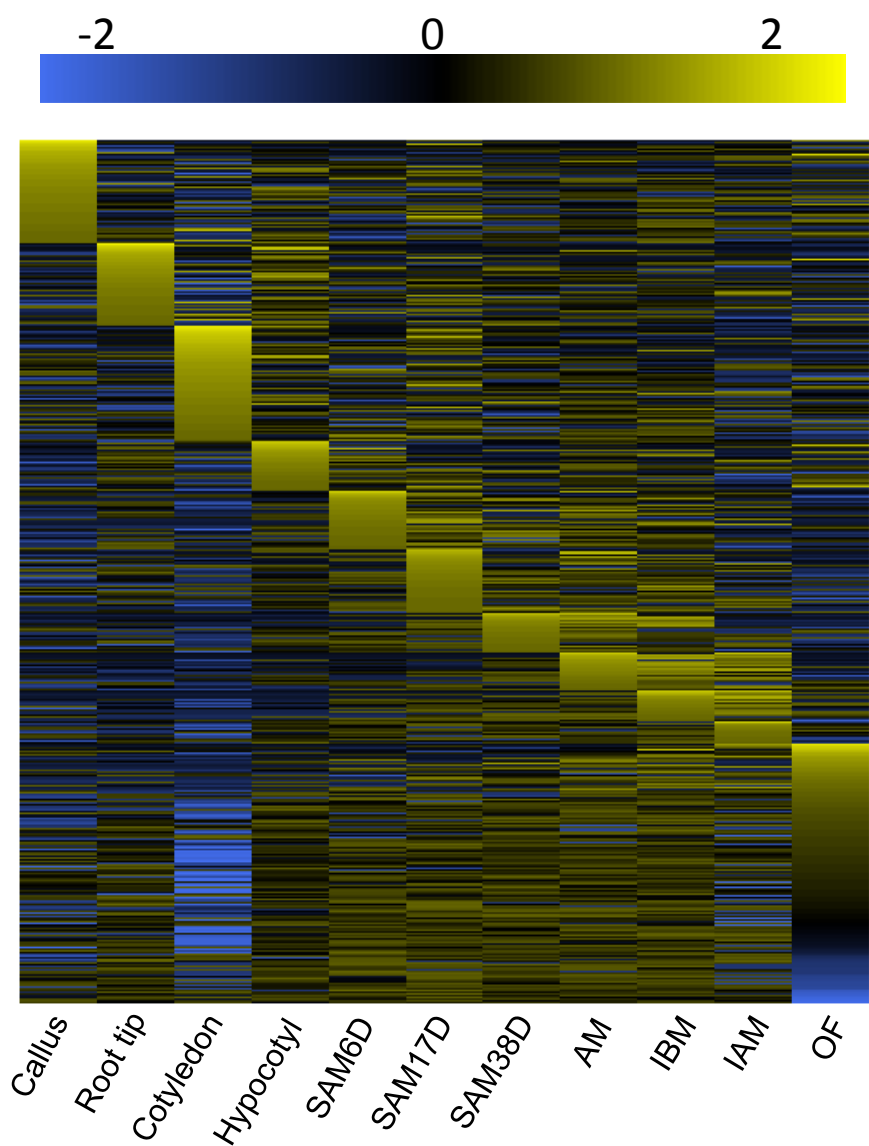

Figure S4

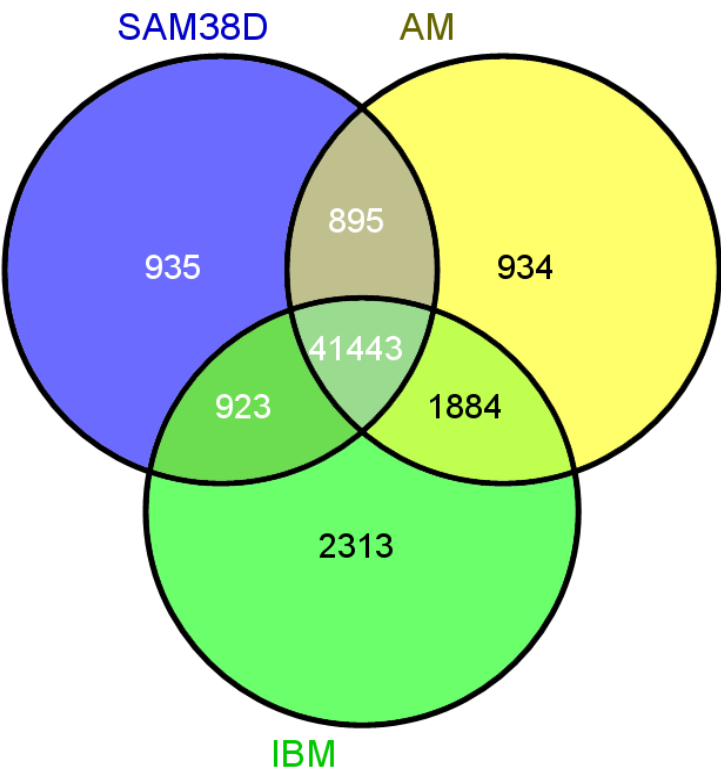

## FigureS5

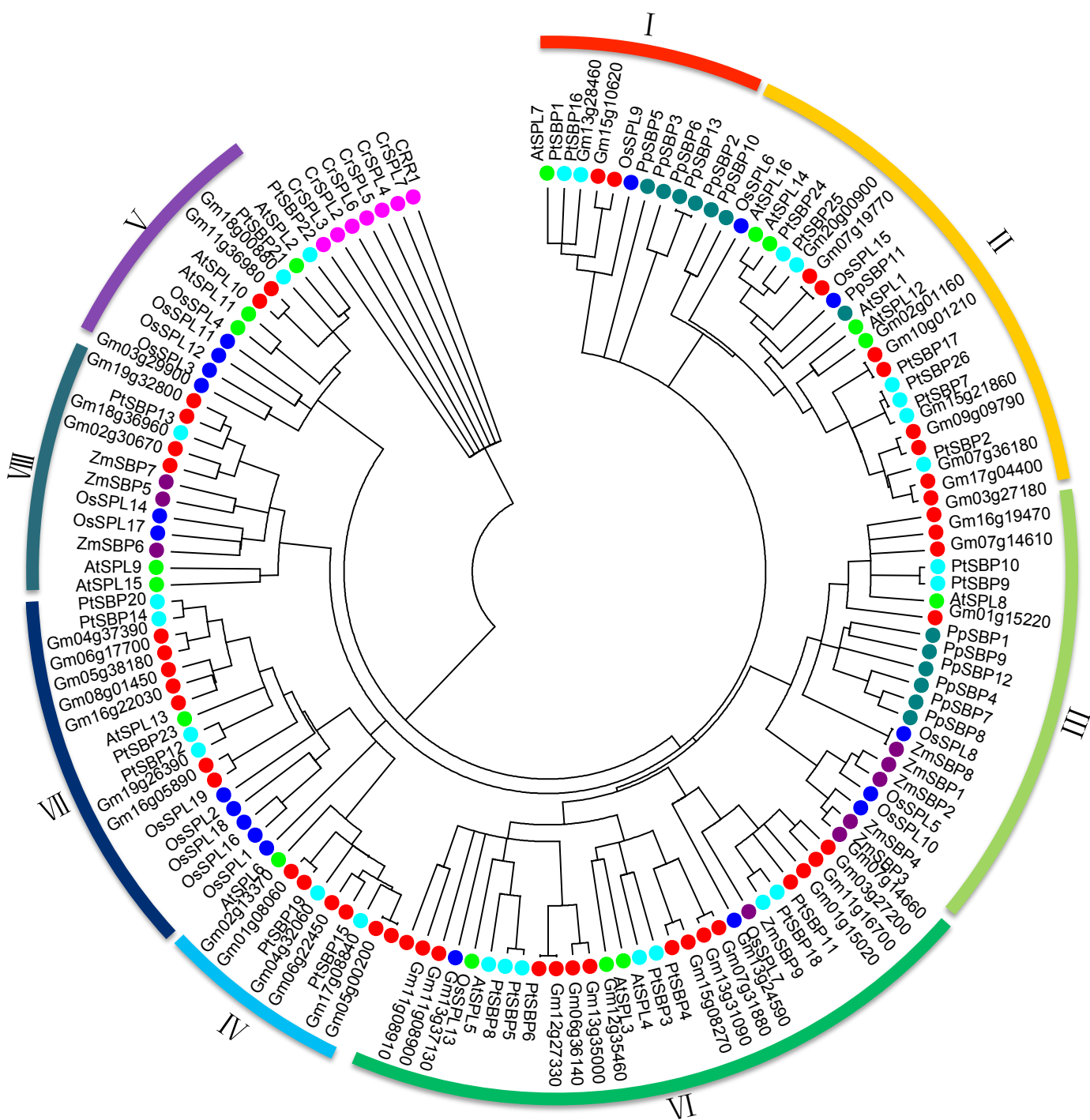

Supplement: Additional file 1: Figure S1 — Total number of reads mapped in samples and distribution of reads among soybean annotated genome. Figure S2. The distribution of RPKM values across 11 samples. (a) Comparison the expression level of genes (blue) and intergenic background regions (red) across 11 soybean tissues. We zoomed in the effects at expression spanning between -15 to 15 log2-transformed RPKM values. (b) The distribution of log2-transformed RPKM values across 11 samples. The vertical dashed line denotes the threshold above which the genes were determined as expressed. The log2-transformed RPKM values of genes at each sample were binned with interval size 0.1. Figure S3. The expression profile of the 4,949 NTUs. Figure S4. Comparison of the expressed genes among SAM38D, AM and IBM. Figure S5. Unrooted phylogenetic tree of the SBP-box family genes based on AA sequences of SBP domains. [file 1471-2229-14-169-S1.pdf]
